# Census Bulletin No. 65

**Published:** 1881-03

**Authors:** 


[Tenth Census of the United States.]

### CENSUS BULLETIN No. 65.

DEPARTMENT OF THE INTERIOR,
CENSUS OFFICE, WASHINGTON, D. C., January 15, 1881.

The following statement exhibits the results of the first count of population according to the schedules returned to the Census Office by the enumerators of the several districts concerned.

The statement of the population in relation to any township, town, city, or county is still subject to possible corrections by reason of the discovery of omissions or duplications of names in lists of inhabitants returned.

### ILLINOIS—(IN PART.)

| COUNTIES. | Total. | Male. | Female. | Native. | Foreign. | White. | Colored.* |
|---|---|---|---|---|---|---|---|
| Alexander | 14,809 | 7,843 | 6,966 | 13,552 | 1,257 | 10,241 | 4,568 |
| Bureau | 33,189 | 17,100 | 16,089 | 27,000 | 6,189 | 33,033 | 156 |
| Champaign | 40,870 | 21,404 | 19,466 | 36,219 | 4,651 | 40,401 | 469 |
| Clark | 21,900 | 11,282 | 10,618 | 21,171 | 729 | 21,849 | 51 |
| Coles | 27,055 | 13,883 | 13,172 | 25,913 | 1,142 | 26,779 | 276 |
| Cumberland | 13,762 | 7,127 | 6,635 | 13,481 | 281 | 13,760 | 2 |
| DeKalb | 26,774 | 13,734 | 12,984 | 21,501 | 5,273 | 26,680 | 94 |
| DeWitt | 17,014 | 8,907 | 8,107 | 16,068 | 946 | 16,901 | 113 |
| Douglas | 15,857 | 8,268 | 7,589 | 15,209 | 648 | 15,747 | 110 |
| Edgar | 25,504 | 13,234 | 12,270 | 24,712 | 792 | 25,322 | 182 |
| Edwards | 8,600 | 4,470 | 4,130 | 7,761 | 839 | 8,517 | 83 |
| Ford | 15,105 | 8,131 | 6,974 | 11,769 | 3,336 | 14,995 | 110 |
| Franklin | 16,129 | 8,268 | 7,861 | 15,985 | 144 | 16,105 | 24 |
| Gallatin | 12,862 | 6,676 | 6,186 | 12,488 | 374 | 12,187 | 675 |
| Hamilton | 16,712 | 8,519 | 8,193 | 16,449 | 263 | 16,669 | 43 |
| Hancock | 35,354 | 18,048 | 17,306 | 31,905 | 3,449 | 35,196 | 158 |

| | | | | | | | |
|---|---|---|---|---|---|---|---|
| Hardin | 6,024 | 3,129 | 2,895 | 5,884 | 140 | 5,860 | 164 |
| Henderson | 10,755 | 5,632 | 5,123 | 9,647 | 1,108 | 10,743 | 12 |
| Henry | 36,610 | 18,907 | 17,703 | 26,949 | 9,661 | 36,478 | 132 |
| Iroquois | 35,457 | 18,780 | 16,677 | 29,006 | 6,451 | 35,209 | 248 |
| Jackson | 22,508 | 11,736 | 10,772 | 20,924 | 1,584 | 20,979 | 1,529 |
| Jasper | 14,515 | 7,515 | 7,000 | 13,839 | 676 | 14,467 | 48 |
| Jefferson | 20,686 | 10,529 | 10,157 | 20,065 | 621 | 20,590 | 96 |
| Jo Daviess | 27,535 | 13,920 | 13,615 | 21,040 | 6,495 | 27,472 | 63 |
| Johnson | 13,079 | 6,676 | 6,403 | 12,961 | 118 | 12,946 | 133 |
| Kankakee | 25,050 | 13,032 | 12,018 | 18,797 | 6,253 | 24,973 | 77 |
| Knox | 38,360 | 19,437 | 18,923 | 31,734 | 6,626 | 37,382 | 978 |
| LaSalle | 70,420 | 36,164 | 34,256 | 52,582 | 17,838 | 70,331 | 89 |
| Lawrence | 13,663 | 7,057 | 6,606 | 13,383 | 280 | 13,345 | 318 |
| Lee | 27,494 | 14,217 | 13,277 | 22,347 | 5,147 | 27,430 | 64 |
| Livingston | 38,453 | 20,401 | 18,052 | 31,225 | 7,228 | 38,153 | 300 |
| McDonough | 27,985 | 14,249 | 13,736 | 26,412 | 1,573 | 27,840 | 145 |
| McHenry | 24,914 | 12,798 | 12,116 | 20,067 | 4,847 | 24,863 | 41 |
| McLean | 60,115 | 30,971 | 29,144 | 52,404 | 7,711 | 59,432 | 683 |
| Macon | 30,672 | 15,953 | 14,719 | 28,212 | 2,460 | 30,315 | 357 |
| Madison | 50,141 | 26,147 | 23,994 | 38,518 | 11,623 | 47,442 | 2,699 |
| Massac | 10,443 | 5,320 | 5,123 | 9,900 | 543 | 8,740 | 1,703 |
| Mercer | 19,505 | 10,127 | 9,378 | 16,964 | 2,541 | 19,468 | 37 |
| Monroe | 13,682 | 7,255 | 6,427 | 10,497 | 3,185 | 13,626 | 56 |
| Moultrie | 13,705 | 7,102 | 6,603 | 13,269 | 436 | 13,689 | 16 |
| Peoria | 55,427 | 28,225 | 27,202 | 44,527 | 10,900 | 54,904 | 523 |
| Perry | 16,008 | 8,242 | 7,766 | 13,985 | 2,023 | 15,230 | 778 |
| Piatt | 15,583 | 8,280 | 7,303 | 14,717 | 866 | 15,550 | 33 |
| Pope | 13,256 | 6,707 | 6,549 | 12,866 | 390 | 12,652 | 604 |
| Pulaski | 9,507 | 4,860 | 4,647 | 9,146 | 361 | 6,238 | 3,269 |
| Randolph | 25,691 | 13,364 | 12,327 | 21,886 | 3,805 | 24,393 | 1,298 |
| Rock Island | 38,315 | 19,755 | 18,560 | 27,914 | 10,401 | 37,774 | 541 |
| Saline | 15,940 | 8,093 | 7,847 | 15,809 | 131 | 15,356 | 584 |
| Shelby | 30,282 | 15,635 | 14,647 | 28,529 | 1,753 | 30,205 | 77 |
| Stark | 11,209 | 5,855 | 5,354 | 10,010 | 1,199 | 11,205 | 4 |
| Tazewell | 29,679 | 15,343 | 14,336 | 24,674 | 5,005 | 29,587 | 92 |

CENSUS BULLETIN NO. 65.—*Continued.*

| COUNTIES. | Total. | Male. | Female. | Native. | Foreign. | White. | Colored* |
|---|---|---|---|---|---|---|---|
| Union | 18,102 | 9,158 | 8,944 | 17,428 | 674 | 17,833 | 269 |
| Vermillion | 41,601 | 21,587 | 20,014 | 38,605 | 2,996 | 41,400 | 201 |
| Wabash | 9,945 | 5,098 | 4,847 | 9,471 | 474 | 9,888 | 57 |
| Washington | 21,117 | 10,922 | 10,195 | 16,852 | 4,265 | 20,905 | 212 |
| Wayne | 21,297 | 10,791 | 10,506 | 20,979 | 318 | 21,283 | 14 |
| White | 23,089 | 12,000 | 11,089 | 22,386 | 703 | 22,556 | 533 |
| Whitesides | 30,888 | 15,887 | 15,001 | 26,048 | 4,840 | 30,810 | 78 |
| Will | 53,431 | 28,447 | 24,984 | 37,256 | 16,175 | 52,727 | 704 |
| Williamson | 19,326 | 9,902 | 9,424 | 19,013 | 313 | 19,073 | 253 |
| Woodford | 21,630 | 11,154 | 10,476 | 17,522 | 4,108 | 21,547 | 83 |

*Including, in Alexander Co., 1 Indian; in Bureau Co., 1 Indian; in Champaign Co., 4 Indians; in Coles Co., 1 Chinese; in Edgar Co., 1 Chinese; in Iroquois Co., 1 Indian; in Jackson Co., 1 Indian; in Jo Daviess Co., 1 Japanese and 6 Indians and Half-Breeds; in Kankakee Co., 2 Chinese; in Knox Co., 2 Chinese and 2 Indians; in LaSalle Co., 1 Chinese; in McDonough Co., 1 Indian; in McLean Co., 4 Chinese; in Macon Co., 3 Chinese and 2 Indians; in Madison Co., 1 Chinese and 2 Indians; in Perry Co., 10 Indians; in Peoria Co., 12 Chinese; in Pope Co., 5 Chinese and 29 Indians: in Rock Island Co., 2 Chinese and 2 Indians; in Tazewell Co., 1 Chinese and 2 Indians; in Vermillion Co., 2 Chinese.

